# Research and Remedies

**Published:** 1912-11-23

**Authors:** 


### RESEARCH AND REMEDIES.

### "Giant Cell Sarcoma."

Opinion in surgical circles has long been veering round to the idea that the malignancy of the tumour called "giant cell sarcoma" is low. For some time local resection of the affected region has been gaining ground, and primary amputation correspondingly losing favour. Now Bloodgood, in the *Annals of Surgery* pleads for a revision, not only of treatment but also of nomenclature. He thinks these growths are not malignant at all, and that the name "sarcoma" should accordingly be dropped in favour of "tumour." He says that conservative treatment is justifiable and preferable to anything radical, and that in many situations curetting should be the operation of choice. Where resection does not interfere with function, as for instance in the upper end of the fibula and the lower end of the ulna, he advises it; but, otherwise, curetting should be given a thorough trial, for it has been known to succeed even when the whole lower end of the femur has been involved. Among twenty-six cases curetted there were only five recurrences; one of these was cured by a second curetting, three by resection, and one by amputation. After curetting or resection the wound should be disinfected with pure carbolic acid, followed by alcohol or chloride of zinc solution. The operation should always be done with a tourniquet when possible. It is not necessary to perform bone transplantation at the primary operation unless a single bone, like the humerus or femur, has been divided in its continuity. Lastly, he deals with problems of diagnosis. It is very important that whenever the $x$-rays disclose a medullary growth the urine should be examined for Bence-Jones bodies, which indicate myeloma or metastatic carcinoma. Bone cysts and giant cell tumours give exactly similar appearances on an $x$-ray plate, and can only be safely differentiated by exploratory operation.

